# Exploring Self-Perceived Stress and Anxiety Throughout Pregnancy: A Longitudinal Study

**DOI:** 10.3390/diseases13040121

**Published:** 2025-04-19

**Authors:** Mar Miguel Redondo, Cristina Liebana-Presa, Javier Pérez-Rivera, Cristian Martín-Vázquez, Natalia Calvo-Ayuso, Rubén García-Fernández

**Affiliations:** 1 Centro de Salud Roces Montevil, Gijón, Servicio de Salud del Principado de Asturias, 33001 Asturias, Spain; guel@sespa.es; 2 HeQoL Research Group, Faculty of Health Sciences, Universidad de León, Campus Universitario de Vegazana, 24071 León, Spain; 3 SALBIS Research Group, Faculty of Health Sciences, Universidad de León, Campus Universitario de Vegazana, 24071 León, Spain; 4 HeQoL Research Group, Faculty of Health Sciences, Universidad de León, Campus Universitario de Ponferrada, 24401 Ponferrda, Spain; cmartv@unileon.es; 5 SALBIS Research Group, Faculty of Health Sciences, Universidad de León, Campus Universitario de Ponferrada, 24401 Ponferrda, Spain; ncala@unileon.es (N.C.-A.); rgarcf@unileon.es (R.G.-F.); 6 Nursing Research, Innovation and Development Centre of Lisbon (CIDNUR), Nursing School of Lisbon, 1600-096 Lisbon, Portugal

**Keywords:** anxiety, health, longitudinal studies, pregnancy, stress

## Abstract

Background: Anxiety and stress are common during pregnancy and can impact the health of the pregnant woman and the newborn. There is a lack of research focused on identifying weaknesses that promote equity in the care of pregnant women. The objective of this study was to describe the levels of anxiety and stress during the three trimesters of pregnancy and to compare whether there are differences according to obstetric and gynecological variables. Methods: A descriptive prospective longitudinal and correlational observational study was carried out. Non-probability sampling was carried out with 176 women. The Pregnancy-Related Anxiety Questionnaire and the Perceived Stress Scale were used. Results: The prevalence of anxiety was 23.9%, 17%, and 17.6%, and mean stress scores reached 32.24, 33.02, and 49.74 in the first, second, and third trimesters, respectively. In comparison, without miscarriages, anxiety was higher during the first trimester. In multiparous women who had suffered a miscarriage, anxiety was higher in the first trimester. Conclusions: Anxiety is higher during the first trimester. Mean stress levels are higher during the third trimester compared to the other two trimesters. Care for these vulnerable pregnant women can impact society’s health system and align with the Sustainable Development Goals of Health and Well-being and Gender Equality in others.

## 1. Introduction

The social, psychological, and hormonal changes inherent to pregnancy frequently precipitate mood disturbances in expectant mothers with a notably high prevalence [1,2]. Stress and anxiety are among the most common mental health challenges during this period and are often closely interlinked [2,3].

Concerns about pregnancy and childbirth causing pregnancy-specific anxiety or affecting fetal development are widespread, affecting up to one in five women [4]. However, global prevalence rates significantly vary and are influenced by cultural factors [5,6] or nulliparity [7]. Anxiety has profound implications for the hypothalamic–pituitary–adrenal axis, inducing systemic inflammation and increasing the risk of obstetric complications [8], including recurrent miscarriage [8], hypertensive disorders, and impaired fetal growth [7]. Furthermore, maternal anxiety is associated with cognitive, linguistic, and socio-emotional developmental difficulties in offspring [9,10].

In countries like Japan and the Netherlands, the prevalence of anxiety during pregnancy is relatively low. This can be attributed to strong social support systems, universal access to prenatal care, and specific educational programs for pregnant women. In contrast, in countries with fewer resources, the lack of social support and limited access to medical care can increase the prevalence of anxiety [5].

Similarly, stress is recognized as a prevalent health concern, particularly early in pregnancy [2]. Its adverse effects extend to pregnancy and labor outcomes [11,12], significantly affecting child development. Elevated maternal stress increases cortisol levels, increasing fetal cortisol levels and potentially inducing epigenetic modifications [11,12]. Such alterations heighten the risk of physical conditions such as asthma [13] or congenital heart disease [14] and emotional and affective disorders [15,16].

Women who are pregnant for the first time (nulliparous) tend to experience higher levels of anxiety due to the lack of previous experience and fear of the unknown. In countries with well-established prenatal education programs, these concerns can be mitigated through information and support [8].

Complications such as pre-eclampsia or gestational diabetes can significantly increase levels of anxiety and stress. In countries with robust healthcare systems, early detection and proper management of these complications can act as protective factors [7].

There is a relatively large literature on stress, anxiety, and depressive symptoms during pregnancy and their implication for postpartum depression [17], newborn body composition [18], inflammatory response about social variables [19], delayed development of motor and communication skills during infancy [20], infant mental health [21], and the impact of the effects of stress and anxiety on the development of infant mental health [21].

Despite the prevalence of pregnancy-related psychopathology, existing research is inconsistent [4]. While some studies indicate heightened stress prevalence early in pregnancy [22], often accompanied by increased anxiety [23,24,25,26], there are progressive increases in these conditions across trimesters [27]. The limited availability of longitudinal studies [6] underscores the need for research that elucidates these dynamics, enabling tailored interventions [28]. Such efforts could promote a balanced maternal neuroendocrine system, improving obstetric and neonatal outcomes [9,29]. Understanding the health status in this group of women allows for the identification of factors that may affect their physical and psychological well-being, the implications this may have on the health of the newborn [30], as well as the promotion of well-being and gender equity in the care of pregnant women.

The COVID-19 pandemic has significantly impacted the mental health of pregnant and postpartum women. A study conducted in Italy during the three years of the pandemic revealed that women who gave birth without their partner’s presence experienced higher levels of depression and post-traumatic stress, as well as lower psychological well-being [31]. Additionally, the psychological burden related to the pandemic and individual coping strategies influenced the risk of developing postpartum depressive symptoms [32]. The lack of support and forced quarantine increased feelings of fatigue and isolation, exacerbating anxiety and depression symptoms in pregnant and postpartum women [33].

This study aims to explore and analyze prenatal anxiety and stress according to obstetric and gynecological factors in the different trimesters of pregnancy.

## 2. Materials and Methods

A longitudinal prospective correlational study was conducted using non-probabilistic convenience sampling. Initially, 466 pregnant women from a health area in the north of Spain were contacted in the first trimester. A total of 360 pregnant women responded to the questionnaires, comprising 77.25% of the total women who were contacted. As this was a longitudinal study, participation was lost in the second and third trimesters, and the final participation comprised 176 pregnant women. The inclusion criteria were (i) to be before the 10th week of gestation; (ii) to be of legal age; (iii) to have a low-risk pregnancy; and (iv) to give informed consent to participate in the study. Exclusion criteria included (i) the presence of medical or psychological disorders limiting their ability to understand this research and the questions in the questionnaires; (ii) when the pregnancy is considered high risk by the Spanish Society of Gynecology; and (iii) being under 18 (legal age in Spain). Participants ranged in age from 20 to 44 years, with a mean of 34.19 ± 4.49 years. Socio-demographic and obstetric–gynecologic characteristics are described in Table 1.

In 2022, 484 births were registered in the region analyzed (Junta de Castilla y León, Spain, 2022) [34]. For this study, the minimum necessary sample size was calculated using a formula based on the population proportion [35], assuming a confidence level of 95% and a margin of error (d) of 3%. As a result, it was determined that 143 participants were needed for the sample.

### 2.1. Instruments

The data collection instruments included a personal information form created by the researchers with obstetric–gynecologic and socio-demographic variables and the validated pregnancy-related anxiety and stress questionnaires.

The Pregnancy-Related Anxiety Questionnaire (PRAQ-20) [24], validated in Spanish, consists of 20 items grouped into five factors: (i) concerns about changes in oneself; (ii) fear for the baby’s integrity; (iii) feelings about oneself; (iv) fear of childbirth; and (v) concerns about the future. Each item is scored on a Likert-type scale. With five response options ranging from 1 (strongly disagree) to 5 (strongly agree), the total score ranges from 20 to 100. The cut-off point of the scale is 67 (85th percentile); women scoring at or above this level will show a high level of gestational anxiety. This tool is considered helpful for measuring anxiety in pregnancy in both multiparous and nulliparous women. The reliability of this PRAQ-20 scale in Spanish [24] was 0.91 during the first and third trimesters and 0.93 during the second and third.

The Perceived Stress Scale (PSS) [36] is a scale validated in Spanish that measures the degree to which everyday situations are stressful, considering feelings and thoughts during the previous month. The Spanish version consists of 14 items, each scored on a Likert-type scale of 0 to 4 (0 = never, 4 = always). The minimum score is 0, and the maximum score is 56. This scale demonstrated reliability, validity, and sensitivity with a Cronbach’s alpha of 0.81 in its validation for the Spanish population [36].

### 2.2. Procedure

The pregnant women participating in this study were recruited in the first trimester of gestation. After the first routine obstetric control visit at the reference hospital of the Health Care Management of a region located in the north of Spain, the researchers individually informed the pregnant women about the present study in a consultation lasting no more than 5 min. If the pregnant woman agreed to participate, after reading and signing the informed consent form, she was asked for her e-mail address or telephone number to be contacted by e-mail or Whatsapp^®^, respectively, and to send the corresponding forms prepared for this study using Google Forms^®^. In total, the pregnant women were approached on three different occasions: the first before 10 weeks of gestation (first trimester), the second after 12 weeks (second trimester), and the third after 36 weeks (third trimester). The average response time for the initial questionnaire was 15 min, while the average response time for the subsequent questionnaires was 10 min. Participants did not receive any compensation for participation in this study. Data were collected between September 2021 and March 2023.

### 2.3. Data Analysis

All data obtained were processed and analyzed using SPSS Statistics V28.0 (IMB. Armonk, NY, USA). In the descriptive analysis of the variables, the frequency of central tendency and dispersion measures were used. The repeated models ANOVA statistical test with Bonferroni correction was performed to compare the means of the presence of stress (PSS) and anxiety (PRAQ-20). This statistical test was also used to compare these variables under study according to different obstetric–gynecologic variables (parity, type of conception, and number of miscarriages). The partial *eta* squared test was used to measure the effect size. *p*-values of less than 0.05 were considered significant. Cronbach’s coefficient was also used for the psychometric analysis of the reliability of the PSS and PRAQ-20 scales.

### 2.4. Ethical Considerations

This study was reviewed and approved by the Institutional Ethics Committee of a Spanish public university (ETICA-ULE-033-2021) and had a favorable report from the Research Ethics Committee of the Health Areas of the northern region of Spain (internal registry 21124), where the pregnant women were attended. This study complies with the international research recommendations proposed by the Declaration of Helsinki, the Belmont Report, and the Oviedo Convention. Organic Law 3/2018 of 5 December on Personal Data Protection and Guarantee of Digital Rights was applied. Participants gave their individual written informed consent to participate in this study.

## 3. Results

**Figure 1:**
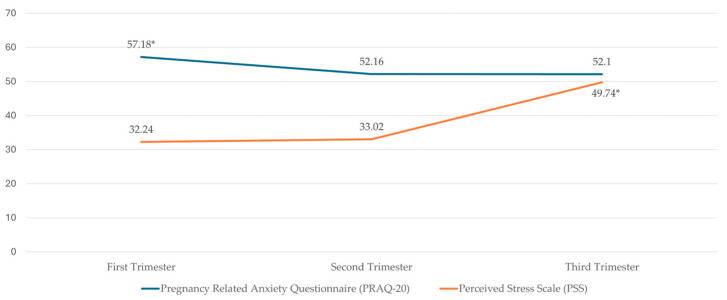
Descriptive statistics of PRAQ-20 and PSS by trimesters * *p* > 0.05.

The prevalence of anxiety was 23.9% in the first trimester, 17% in the second trimester, and 17.6% in the third trimester of gestation. In addition, the mean values of anxiety and stress in the different trimesters of gestation are shown in Figure 1.

Table 2 shows the psychometric analyses of the reliability of the questionnaires used, PSS and PRAQ-20, and the dimensions of the latter questionnaire.

In Table 3, descriptive statistics and mean differences of anxiety and stress variables are detailed according to the gestational trimester. Stress scores were statistically higher in the third trimester compared to the first and second trimesters. However, regarding total anxiety, women in the first trimester of pregnancy had higher values than those in the third and second trimesters. Specifically, concerning the dimensions of the PRAQ-20, it is noteworthy that concern about changes in oneself and fear for the baby’s integrity were statistically higher in the first trimester than in the other two trimesters.

The results showed statistically significant differences between women who had one or more previous abortions and those who had no previous abortions. Third-trimester women with previous abortions (57.05 ± 15.18) had a higher level of anxiety than those who did not have previous abortions (50.01 ± 15.76), (t (174) = −2.73; *p* = 0.003).

Table 4 describes the results achieved for stress and anxiety levels according to parity and trimester of gestation. Thus, in primiparous women, the anxiety score is statistically higher in the first trimester than in the third and second trimesters. Worries about changes in oneself, fear for the baby’s integrity, and fear of childbirth follow the same trend. Regarding stress, first-time pregnant women showed higher values in the third trimester than in the first and second trimesters. On the other hand, in multiparous pregnant women, anxiety was higher in the first trimester than in the second; in particular, the dimensions of fear of childbirth also follow this trend. Stress is still higher in the third trimester than in the first and second trimesters in women who have been previously pregnant.

Regarding the results obtained for stress and anxiety levels according to the number of miscarriages and trimester of gestation in women who had not experienced any miscarriage, the anxiety score was statistically higher in the first trimester than in the third.

Concerning stress in women who have not experienced miscarriages, the score was statistically higher during the third trimester than during the first and second trimesters. On the other hand, in women who have experienced one miscarriage, anxiety has not experienced a significant variation across trimesters. At the same time, stress remains higher in the third trimester than in the second and first trimesters. In women who had experienced two or more miscarriages, anxiety again showed no significant variation. At the same time, stress followed the same trend, which was higher in the third trimester than in the second and first trimesters.

The results were obtained for stress and anxiety levels according to the type of conception and trimester of gestation. In women with spontaneous pregnancies, the anxiety score was statistically higher in the first trimester than in the second and third trimesters. For stress, the score was statistically higher in the third trimester than in the first and second trimesters. In women with assisted reproduction pregnancies, anxiety, and stress scores did not significantly vary between trimesters.

## 4. Discussion

The results obtained show the self-perceived levels of anxiety and stress across the three trimesters of pregnancy and help analyze the relationship between these variables. They also compare differences in anxiety and stress based on obstetric–gynecologic variables and the trimester of pregnancy.

The PRAQ-20 scale shows high internal consistency, with values like those obtained in the Spanish validation study [24] above 0.90 in all trimesters. It was found that the prevalence of anxiety was statistically higher during the first trimester, being present in 23.9% of the participants. It was 17% and 17.6% during the second and third trimesters. This decreasing evolution of the percentage of anxiety with a slight increase during the third trimester, although not significant for this study, is like that previously obtained by other authors [24,26]. When different versions of the PRAQ-20 with fewer items are used, such as the PRAQ-20, mean anxiety values slightly lower than those in our study are obtained [28,37].

As in previous studies, the different dimensions of PRAQ-20 show significant variations throughout trimesters [28]. In our study, fear for the baby’s integrity exhibited the strongest positive correlations with the presence of anxiety in each trimester, followed by fear of childbirth. These findings are consistent with previous research, which indicates that such worries are closely linked to significant concerns during pregnancy. These concerns include factors that may affect maternal and fetal health, potential complications during childbirth, and social and economic support levels, among others [38]. Based on these results, it may be interesting for future studies to evaluate the total anxiety score and continue studying the different dimensions that influence its presence.

The PSS scale also demonstrates high internal consistency. with values exceeding those reported in the Spanish validation study [36]. It reaches values above 0.90, except in the first trimester, where it is 0.88. In our study, stress significantly increased during the third trimester, with mean values of 32.24, 33.02, and 49.74 for the first, second, and third trimesters, respectively. This progression aligns with recent longitudinal studies conducted during the COVID-19 pandemic [27] but contrasts with pre-pandemic studies where stress was highest during the first trimester and subsequently decreased [22]. Another aspect to consider is that the mean stress levels reported in studies are generally lower than in our research [39,40,41]. On the other hand, some studies identified stress levels in the first trimester during the pandemic similar to those observed in our study. Although the samples in these studies differ, the target population remains the same [42].

The relationship between anxiety and stress in our study differs from what has been previously proposed by other authors [22,43]. However, following the COVID-19 pandemic, longitudinal studies have suggested that the evolution of both variables is similar to our study findings [27]. Despite the differing trends in both variables, specific correlations were consistent with those in prior research [43]. In our study, first-trimester stress positively correlates with anxiety in the first and second trimesters. This aligns with other studies that, despite identifying elevated stress and lower anxiety levels in the first trimester, observed that the presence of anxiety symptoms increased the risk of stress and that stress acted as a risk factor for the development of anxiety [44]. Additionally, in our study, the presence of anxiety during pregnancy is associated with stress at the end of pregnancy. The dimension of anxiety most strongly correlated with third-trimester stress involves self-related concerns such as weight gain, which aligns with the increased correlation observed at the end of pregnancy, a period when pregnant women reach their maximum weight [45].

Our study’s sample was homogeneous regarding gravidity: 52.8% were experiencing their first pregnancy, while 47.2% had been pregnant before. Some studies suggest multiparous women experience less stress during pregnancy [46,47]. However, in our study, both groups exhibited statistically higher stress levels during the third trimester. Regarding anxiety, previous studies indicate that primiparous women have higher levels of anxiety [2,47,48]. In our study, anxiety was significantly higher during the first trimester for both groups. However, in women who had been pregnant before [49], anxiety slightly increased again during the third trimester. This may be related to concerns about the baby’s well-being. as anxiety tends to slightly rise at the end of pregnancy in women with previous pregnancies. This situation could stem from the approach to childbirth, particularly in women with a personal history of obstetric complications in earlier deliveries. It would be valuable to explore both parity and the nature of those past experiences for future studies. As for the fear of childbirth, one of the most prevalent dimensions of anxiety in our study, it remained stable among multiparous women; in comparison, it was statistically higher for primiparous women during the first trimester. It slightly increased again during the third trimester, likely due to the impending childbirth for which they lack prior experience. Certain studies suggest that fear of childbirth is generally greater in primiparous women [38], but a recent systematic review indicates that both groups may experience similar levels of anxiety [49].

Regarding women with a history of miscarriages, most participants in our study (70.5%) had not experienced any previous miscarriage, 23.3% had experienced one miscarriage, and 6.3% had experienced two or more. Previous studies indicate that stress and anxiety are more frequent in women who have suffered recurrent miscarriages [10,50]. In our study, stress levels were statistically higher during the third trimester across all groups. Anxiety levels, however, varied depending on the number of miscarriages. Women with two or more miscarriages maintained elevated anxiety levels throughout the entire pregnancy with no variability based on the trimester. In contrast, women with no prior miscarriages experienced the highest levels of anxiety during the first trimester, followed by a decline, possibly due to increased confidence in the pregnancy’s progress [28]. Women who had experienced only one miscarriage showed a renewed increase in anxiety during the third trimester.

In our study, the sample was heterogeneous in terms of conception method, with only 5.7% of the women having undergone assisted reproductive techniques. Previous studies have associated this type of conception with reduced stress levels [47]. However, in our study, both groups had higher stress levels during the third trimester.

This study has several limitations. Firstly, the participants are low-risk pregnant women, which may limit the generalizability of the results. Women with high-risk pregnancies may experience different patterns of stress and anxiety due to additional concerns. Secondly, this study did not account for the presence of stressful life events unrelated to pregnancy or other factors such as social support, employment status, associated psychiatric disorders, or pre-existing health conditions. However, this longitudinal salutogenic approach aims to reaffirm the physiological nature of pregnancy, thereby providing a foundation for future, more rigorous, and precise studies to document and address the needs of various subgroups. It also highlights the importance of a preventive and health-promoting approach for this population. Furthermore, recent studies should be designed using longitudinal methodology, uncomplicating comparisons.

Although it is difficult to determine whether stress precedes anxiety or vice versa, our findings suggest that early interventions aimed at reducing anxiety may have a positive effect on the subsequent reduction in stress, thereby improving maternal and neonatal outcomes.

This longitudinal study provides a comprehensive overview of the evolution of anxiety and stress throughout the three trimesters of pregnancy. Understanding how these two variables develop over time is crucial for designing more targeted and specific interventions, protocols, and programs that address the holistic needs of pregnant women. Pregnancy is a physiological process, so our proposed approach emphasizes health promotion and disease prevention.

These interventions should be delivered primarily through primary healthcare, led by nurses specialized in obstetrics and gynecology or by nurses specialized in family or community health. This would enhance access to the healthcare system while reducing unnecessary medicalization.

It is, therefore, recommended that more comprehensive prenatal education programs be implemented, with a strong focus on the emotional and mental health needs of pregnant women. Such programs could help reduce anxiety and stress, leading to improved maternal and fetal health outcomes.

Furthermore, providing psychological and emotional support is essential. Pregnant women should be equipped with tools to manage this period effectively, taking into account the biopsychosocial factors influencing their well-being and promoting a healthier pregnancy overall.

Implementing an early detection and management program for gestational complications is crucial. Early identification and management of complications can alleviate anxiety and stress, thus improving obstetric outcomes.

Finally, developing and implementing social and economic support policies are critical to reducing stress related to financial concerns and access to healthcare, ultimately contributing to the well-being of both mother and fetus.

## 5. Conclusions

Pregnancy-specific anxiety was higher in the participating pregnant women during the first trimester compared to the other two trimesters. Specifically, 23.9% exhibited elevated levels of this variable in the first trimester. Conversely, perceived stress among pregnant women was higher in the third trimester as the moment of childbirth approached.

In primiparous women, those who have never experienced a miscarriage or those with a spontaneous conception, anxiety remains higher in the first trimester. The most concerning factors during this period include self-focused worries, fear for the baby’s health, and fear of childbirth. The stress variable, however, continues to be higher in the final trimester regardless of the parity, type of conception, or number of miscarriages.

Effective interventions within prenatal health education programs that focus on reducing anxiety at the beginning of pregnancy and managing emotional stress toward the end could help alleviate concerns and improve pregnant women’s perception of their health. Tailored interventions based on the type of parity or number of miscarriages could positively impact the quality of care received. Furthermore, care for this vulnerable group of pregnant women can affect the health system and society and align with the Sustainable Development Goals of Health and Wellbeing and Gender Equality in others.

Based on the study results, we recommend implementing prenatal education programs with stress- and anxiety-management techniques for the first trimester. Additionally, emotional and psychological support sessions should be offered, especially for primiparous women and those with a history of spontaneous abortions. These interventions can improve pregnant women’s health perception and maternal and neonatal health outcomes.

## Figures and Tables

**Table 1:** Socio-demographic and obstetric–gynecologic characteristics of participants.

Variables		n 176 (100%)
Marital status	Married/inhabiting	140 (79.5%)
	single/widowed	36 (20.5%)
Educational level	University education	72 (40.9%)
	Postgraduate	24 (13.6%)
	Primary education	4 (2.3%)
	Secondary education	76 (43.2%)
Nationality	Spanish	170 (96.6%)
	Other	6 (3.4%)
Area of residence	Rural	60 (34.1%)
	Urban	116 (65.9%)
Parity	One	93 (52.8%)
	Two or more	83 (47.2%)
Abortions	Zero	124 (70.5%)
	One	41 (23.3%)
	Two or more	11 (6.3%)
Vaginal births	Zero	127 (72.2%)
	One	45 (25.6%)
	Two or more	4 (2.3%)
Cesarean sections	Zero	148 (84.1)
	One	26 (14.8%)
	Two or more	2 (1.1%)
Conception	Spontaneous	166 (94.3%)
	Assisted reproduction	10 (5.7%)

**Table 2:** Psychometric analysis of the reliability of the PRAQ-20 and PSS.

Variable	*α*
Perceived Stress Scale (PSS)	1st T 0.882
	2nd T 0.954
	3rd T 0.923
Pregnancy-Related Anxiety Questionnaire-20 (PRAQ-20)	1st T 0.909
	2nd T 0.912
	3rd T 0.916
Concern for change in oneself	1st T 0.774
	2nd T 0.896
	3rd T 0.872
Fear for the integrity of the baby	1st T 0.917
	2nd T 0.910
	3rd T 0.919
Feelings about oneself	1st T 0.849
	2nd T 0.834
	3rd T 0.808
Fear of childbirth	1st T 0.822
	2nd T 0.850
	3rd T 0.852
Concern about the future	1st T 0.732
	2nd T 0.645
	3rd T 0.715

*α*: Cronbach’s alpha.

**Table 3:** Descriptive statistics and mean differences by trimester for PRAQ-20 and PSS.

Variable	1st T M(SD)	2nd T M(SD)	3rd T M(SD)	C	M.diff	SD	*p*	$\eta_{p}^{2}$
Perceived Stress Scale (PSS)	32.24 (5.46)	33.02 (4.61)	49.74 (6.48)	1-2	−0.78	0.49	0.334	0.993
				1-3	−17.50 *	0.65	<0.001	
				3-2	16.72 *	0.60	<0.001	
Pregnancy-Related Anxiety Questionnaire-20 (PRAQ-20)	57.18 (15.51)	52.16 (15.49)	52.09 (15.88)	1-2	5.01 *	0.82	<0.001	0.935
				1-3	5.08 *	0.95	<0.001	
				2-3	0.07	0.78	1	
Concern for change in oneself	7.26 (3.32)	6.45 (3.39)	6.25 (3.26)	1-2	0.80 *	0.21	<0.001	0.839
				1-3	1.01 *	0.22	<0.001	
				2-3	0.20	0.19	0.833	
Fear for the integrity of the baby	27.9 (7.31)	24.6 (7.66)	24.57 (7.89)	1-2	3.29 *	0.43	<0.001	0.934
				1-3	3.33 *	0.48	<0.001	
				2-3	0.04	0.38	1	
Feelings about oneself	6.75 (3.32)	6.51 (3.00)	6.32 (3.02)	1-2	0.24	0.18	0.553	0.850
				1-3	0.43	0.21	0.128	
				2-3	0.18	0.19	1	
Fear of childbirth	10.60 (4.68)	9.99 (4.70)	10.33 (4.82)	1-2	0.62	0.28	0.088	0.861
				1-3	0.28	0.31	1	
				2-3	−0.34	0.29	0.736	
Concern about the future	4.66 (2.35)	4.60 (2.11)	4.63 (2.12)	1-2	0.06	0.15	1	0.860
				1-3	0.04	0.16	1	
				2-3	−0.02	0.14	1	

Abbreviations: 1st T: first trimester; 2nd T: second trimester; 3rd T: third trimester; M(SD): mean (standard deviation); C: trimesterly comparison; M.diff: mean difference; *: *p* < 0.05; $\eta_{p}^{2}$: partial eta squared.

**Table 4:** Descriptive statistics and mean differences by trimester and parity on PRAQ-20 and PSS.

	Variable	1st T M(SD)	2nd T M(SD)	3rd T M(SD)	C	M.diff	SD	*p*	$\eta_{p}^{2}$
Primiparous	Perceived Stress Scale (PSS)	31.71 (5.50)	32.84 (4.72)	50.53 (4.98)	1-2	−1.24	0.72	0.260	
					1-3	−18–82 *	0.80	<0.001	0.994
					3-2	17.58 *	0.67	<0.001	
	Pregnancy-Related Anxiety Questionnaire-20 (PRAQ-20)	57.34 (16.68)	51.68 (14.94)	50.77 (15.68)	1-2	5.67 *	1.16	<0.001	
					1-3	6.57 *	1.36	<0.001	0.934
					3-2	−0.90	1.19	<0.001	
	Concern for changes in oneself	6.89 (3.05)	6.25 (2.86)	5.92 (2.90)	1-2	0.64 *	0.24	0.026	
					1-3	0.97 *	0.28	0.003	0.866
					3-2	−0.32	0.29	0.807	
	Fear for the integrity of the baby	27.41 (7.88)	23.48 (8.00)	22.94 (8.29)	1-2	3.92 *	0.63	<0.001	
					1-3	4.47 *	0.72	<0.001	0.921
					3-2	−0.55	0.56	0.994	
	Feelings about oneself	6.10 (3.05)	6.08 (2.49)	6.04 (2.74)	1-2	0.22	0.24	1	0.872
					1-3	0.05	0.28	1	
					3-2	−0.03	0.27	1	
	Fear of childbirth	12.02 (4.69)	11.00 (4.66)	11.15 (4.78)	1-2	1.01	0.42	0.051	
					1-3	0.86	0.42	0.130	0.888
					3-2	0.15	0.45	1	
	Concern about the future	4.94 (2.46)	4.87 (2.16)	4.72 (2.07)	1-2	0.06	0.23	1	0.873
					1-3	0.21	0.23	1	
					3-2	−0.15	0.21	1	
Multiparous	Perceived Stress Scale (PSS)	32.84 (5.39)	33.11 (4.51)	48.87 (7.76)	1-2	−0.26	0.65	1	
					1-3	−16.02 *	1.03	<0.001	0.991
					3-2	15.76 *	1.02	<0.001	
	Pregnancy-Related Anxiety Questionnaire-20 (PRAQ-20)	56.99 (14.19)	52.71 (16.16)	53.58 (16.07)	1-2	4.23 *	1.15	0.001	
					1-3	3.41 *	1.29	0.030	0.936
					3-2	0.87	0.97	1	
	Concern for changes in oneself	7.66 (3.57)	6.69 (3.91)	6.61 (3.61)	1-2	0.98 *	0.35	0.021	
					1-3	1.05 *	0.35	0.011	0.819
					3-2	−0.07	0.23	1	
	Fear for the integrity of the baby	28.45 (6.62)	25.87 (7.09)	26.40 (7.03)	1-2	2.58 *	0.58	<0.001	
					1-3	2.05 *	0.61	0.004	0.950
					3-2	0.53	0.52	0.921	
	Feelings about oneself	7.48 (3.46)	6.99 (3.46)	6.64 (3.30)	1-2	0.49	0.28	0.237	
					1-3	0.84 *	0.31	0.022	0.842
					3-2	−0.35	0.26	0.526	
	Fear of childbirth	9.04 (4.15)	8.86 (4.51)	9.41 (7.72)	1-2	0.18	0.37	1	0.844
					1-3	−0.37	0.45	1	
					3-2	0.55	0.37	0.404	
	Concern about the future	4.36 (2.20)	4.31 (2.04)	4.52 (2.17)	1-2	0.05	0.76	1	0.847
					1-3	−0.16	0.22	1	
					3-2	0.20	0.18	0.77	

Abbreviations: 1st T: first trimester; 2nd T: second trimester; 3rd T: third trimester; M(SD): mean (standard deviation); C: trimesterly comparison; M.diff: mean difference; *: *p*< 0.05; $\eta_{p}^{2}$: partial eta squared.

## Data Availability

The data analyzed in this study will be made available upon reasonable request by the corresponding author.
